# Clinical characteristics and outcomes of ischemic stroke patients during Ramadan vs. non-Ramadan months: Is there a difference?

**DOI:** 10.3389/fneur.2022.925764

**Published:** 2022-07-22

**Authors:** Naser Alotaibi, Mohammed A. Aldriweesh, Muath A. Alhasson, Bayan A. Albdah, Abdulaziz A. Aldbas, Waleed A. Alluhidan, Sultan A. Alsaif, Faisal M. Almutairi, Mohammed A. Alskaini, Ali M. Al Khathaami

**Affiliations:** ^1^College of Medicine, King Saud Bin Abdulaziz University for Health Sciences, Riyadh, Saudi Arabia; ^2^King Abdullah International Medical Research Center, Riyadh, Saudi Arabia; ^3^Division of Neurology, Department of Medicine, King Abdulaziz Medical City, National Guard Health Affairs, Riyadh, Saudi Arabia; ^4^Unaizah College of Medicine, Qassim University, Qassim, Saudi Arabia; ^5^College of Medicine, Imam Mohammad Ibn Saud Islamic University, Riyadh, Saudi Arabia; ^6^College of Medicine, Almaarefa University, Riyadh, Saudi Arabia; ^7^Department of Neurology, Prince Sultan Military Medical City, Riyadh, Saudi Arabia

**Keywords:** ischemic stroke, Ramadan fasting, risk-factors, frequency, intermittent fasting

## Abstract

**Objectives:**

To study the clinical characteristics and outcomes of patients experiencing an ischemic stroke during Ramadan vs. non-Ramadan months in a tertiary academic center in an Islamic country.

**Methods:**

We retrospectively reviewed all patients with ischemic stroke (IS) in Ramadan and non-Ramadan months for four consecutive years (February 2016–June 2019). All demographics, vascular risk factors, laboratory results, modified Rankin Scale (mRS) at admission and discharge, National Institute Stroke Scale (NIHSS), and in-hospital complication data were collected for all patients.

**Results:**

One thousand and 58 patients were included (non-Ramadan, *n* = 960; during Ramadan, *n* = 98). The mean age during Ramadan was 59 ± 13 years. Most non-Ramadan IS patients during Ramadan were male (68.5%; 57.1%, respectively). There was no statistical difference in vascular risk factors and medical history between the two groups. However, Ramadan patients had higher median NIHSS scores at discharge (*p* = 0.0045). In addition, more ICU admissions were noted among Ramadan patients (*p* = 0.009). In the gender-specific analysis for Ramadan patients, we found a statistically significant difference in smoking and urinary tract infection (*p* = 0.006, *p* = 0.005, respectively).

**Conclusion:**

Based on our results, there was no difference, in general, between patients with IS during Ramadan and non-Ramadan months. However, IS patients had higher NIHSS scores at discharge and more ICU admissions during Ramadan. Last, we suggest future studies with larger sample sizes, longer duration, and including all types of strokes.

## Introduction

During Ramadan, the 9^th^ month of the Islamic calendar, most Muslims worldwide fast for about 18 h or more each day. Fasting is a time for spiritual renewal, contemplation, and prayer. Since Muslims fast around 18 h every day for a month, this comes with a risk of hypoglycemia, dehydration, sleep disturbances, and changes in blood pressure. Most Muslims experience a difference in the quality of food during Ramadan and a shortened period in which they can eat or drink. This is usually associated with a significant alteration in their eating habits (1). As a result, Muslims have different metabolic changes during Ramadan (1). One study showed an improvement in 10-year coronary heart disease risk scores and other cardiovascular risk factors such as weight, body mass index (BMI), and waist circumference after an average of 26 days of fasting during Ramadan (2). Another study showed a significant improvement in lipid profile and blood pressure in normal healthy subjects, patients with stable cardiac illness, metabolic syndrome, dyslipidemia, and hypertension during Ramadan fasting (3). A large study conducted on patients with diabetes showed that fasting in Ramadan positively affects blood glucose and glycated hemoglobin (HbA1c) in the follow-up (4).

On the other hand, a systematic review of 22 studies reported inconsistent findings during Ramadan regarding HbA1c, blood pressure, lipid profile, and BMI, attributed to different dietary habits and physical activities among participants from various studies (5). A study in Israel demonstrated that the incidence of ischemic stroke increased during Ramadan, especially during the first 2 weeks (6). Another study in southwest Turkey revealed that the mean number of patients during Ramadan was 27, similar to the other months (7). Similarly, a study in Qatar showed no difference in stroke incidence during Ramadan (8). Turkish research manifested a significant increase in ischemic stroke among people with diabetes during Ramadan, in contrast to a decrease in intracerebral hemorrhage among hypertensive patients (9). In the Kingdom of Saudi Arabia (KSA), the incidence rate of all strokes ranged between 175.8 and 196.2 per 100,000, and the rate was between 39.7 and 48.6 for intracerebral hemorrhage (10). Also, the incidence rate for ischemic stroke was between 131.0 and 151.5 (10). Looking at the number of strokes in an Islamic country such as KSA reflects the crucial need to understand the association and nature between Ramadan and stroke. Thus, we aimed to better understand stroke during Ramadan, including the frequency of stroke, risk factors, complications, and outcomes.

## Materials and methods

### Study design, area, and settings

Between February 2016 and July 2019, we screened all patients with ischemic stroke or intracerebral hemorrhage admitted to the stroke unit at King Abdulaziz Medical City, Riyadh, Saudi Arabia (KAMC-RYD). We included any patients with a confirmed diagnosis of ischemic stroke; a stroke neurologist confirmed the final diagnosis. Moreover, all included patients had to fulfill the following criteria: (1) sudden neurological deficit resembling stroke within 24 h before arrival to the emergency department; (2) evidence of acute brain infarct or detection of acute brain infarct by brain computed tomography (CT) or magnetic resonance imaging (MRI) corresponding to the neurological deficit. We excluded all patients with hemorrhagic stroke, transient ischemic attack (TIA), traumatic brain hemorrhage, and those older than 80 years of age who were either bedridden, had a prior disabling stroke, dementia, or terminal cancer. Table 1 shows Ramadan months, Islamic Hijri years, and corresponding Gregorian months. The patients were divided into two groups: Non-Ramadan and during Ramadan. We define the “Non-Ramadan” group as patients diagnosed with ischemic stroke in the months before and after Ramadan during the 4 years of the study.

**Table 1:** The Gregorian months corresponding to the Islamic Hijri month Ramadan.

**Year**	**Gregorian Month (s)**	**Islamic Hijri Year**
2016	06/June−05/July	1,437
2017	27/May−24/June	1,438
2018	16/May−14/June	1,439
2019	06/May−04/June	1,440

### Data collection

All medical records for patients who fit the inclusion criteria were reviewed. In addition, the following variables were collected: date of admission, demographic data, vascular risk factors, treatment with reperfusion therapy (t-PA) or endovascular treatment (EVT), modified Rankin scale (mRS) on admission, National Institute of Health Stroke Scale (NIHSS) on admission and discharge, length of stay (LOS), and in-hospital complications and death. In addition, the treating team collected NIHSS, mRS, and in-hospital complications at presentation and during the hospital stay.

### Statistical analysis

Statistical package SAS JMP Pro 15 (SAS Institute Inc., Cary, NC) for Apple Macintosh was used to analyze the data. Means, standard deviations, and medians were used to present continuous variables (interquartile range). Counts and percentages were used to represent categorical variables. The differences between frequencies were investigated using a chi-square test. Continuous variables were tested using the *T*-test, ANOVA, and Kruskal-Wallis test. With two-sided testing, differences were considered statistically significant if the *p*-value was < 0.05.

## Results

The sample size consisted of 1,058 IS patients. They were divided into in non-Ramdan months (*n* = 960) and during Ramadan (*n* = 98). A comparison was made of the two groups, including incidence rate of stroke, mean age, gender variations, risk factors, outcomes, and complications. The division of the sample size into two groups (Ramadan and non-Ramadan) instead of three (Ramadan, before Ramadan, and after Ramadan) was better due to easier interpretations. Also, it was more useful in answering our research question since we aimed to evaluate Ramadan months in comparison to the rest of the year.

There is no significant difference in the mean age between the two groups (*p* = 0.08). However, there are apparent gender differences (*p* = 0.02). Males were more prone to ischemic stroke in non-Ramadan months than females (68.5 and 31.5%, respectively). On the other hand, gender distribution between males and females occurs during Ramadan (57 and 42%, respectively). No difference was noted regarding the risk factors between the two groups. Upon admission, NIHSS scores did not show a difference (*p* = 0.09). However, the mRS score showed a significant difference (*p* = 0.03). Laboratory results upon admission did not vary between the two groups, except for blood urea nitrogen (BUN) and creatinine, both of which had a higher mean in the non-Ramadan group (*p* = 0.0002 and 0.0005, respectively). There was no significant difference in complications between the two groups.

**Figure 1:**
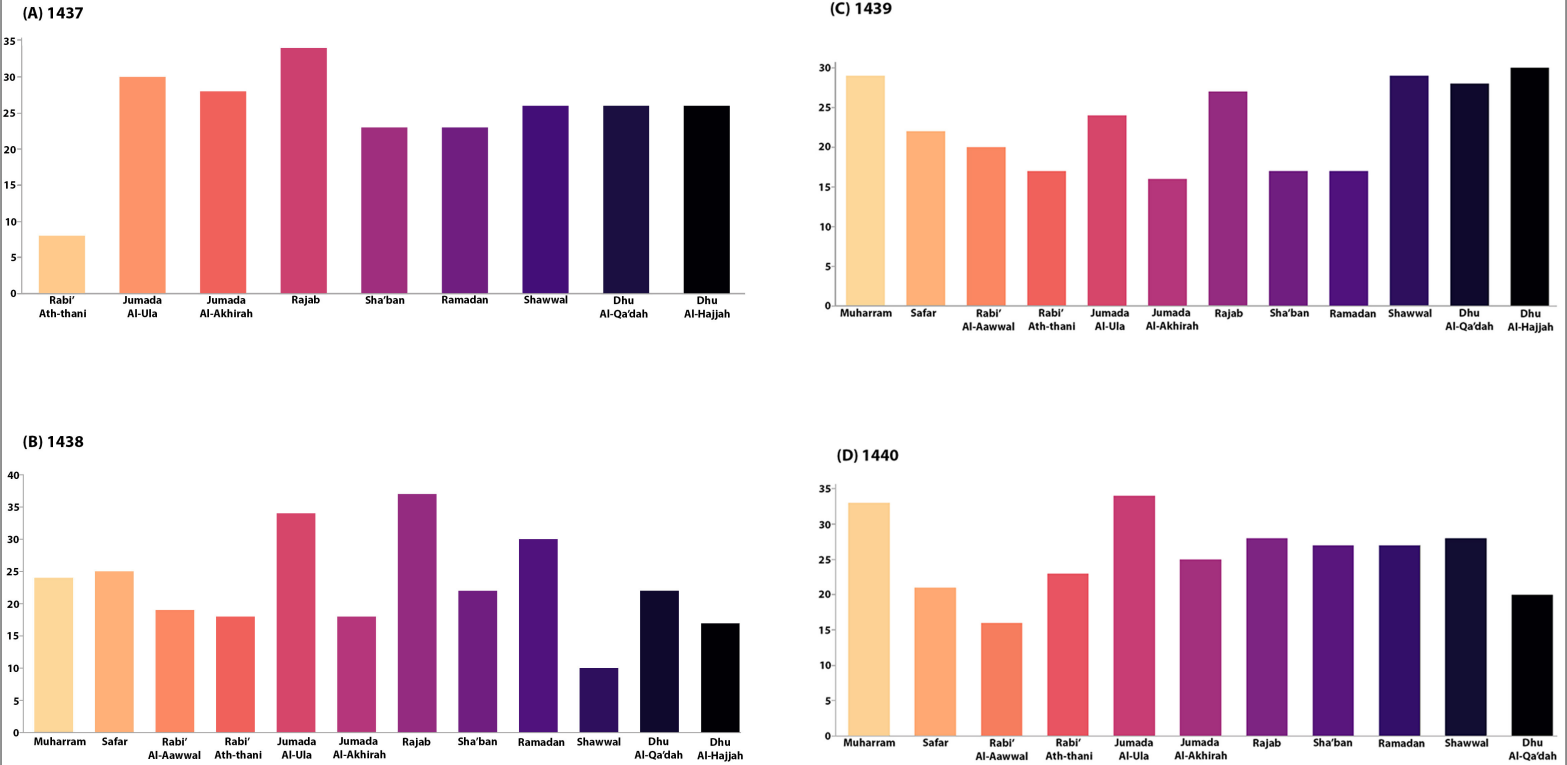
The monthly distribution of Ischemic Stroke (IS) cases is stratified by different months, including Ramadan and non-Ramadan. **(A)** Shows the trend of IS cases per month between Rabi' Ath-Thani 1,437 and Dhu Al-Hijjah 1,437; this corresponds to 06/June – 05/July 2016. A trend is noticed in Rajab, followed by Jumada Al-Aula. However, the trend of cases in Sha'ban and Ramadan was similar. **(B)** Depicts the trend of IS cases per month between Muharram 1,438 and Dhu Al-Hijjah 1,438; this corresponds to 27/May – 24/June 2017. Interestingly, most cases happened during Dhu Al-Hijjah, followed by Shawaal, Muharram. Regarding Ramadan cases, they were almost equal to Sha'ban. **(C)** Demonstrates the trends of IS cases per month between Muharram 1,439 and Dhu Al-Hijjah 1,439; this corresponds to 16/May – 14/June 2018. Many cases occurred during July, followed by September. **(D)** Illustrates the IS cases per month trends between Muharram 1,440 and Dhu Al-Qa'dah 1,440; this corresponds to 06/May – 04/June 2019. Most cases were noted in Jumada Al-Ula, followed by Muharram. Concerning Ramadan, the cases were lower than Sha'ban and Shawaal.

**Figure 2:**
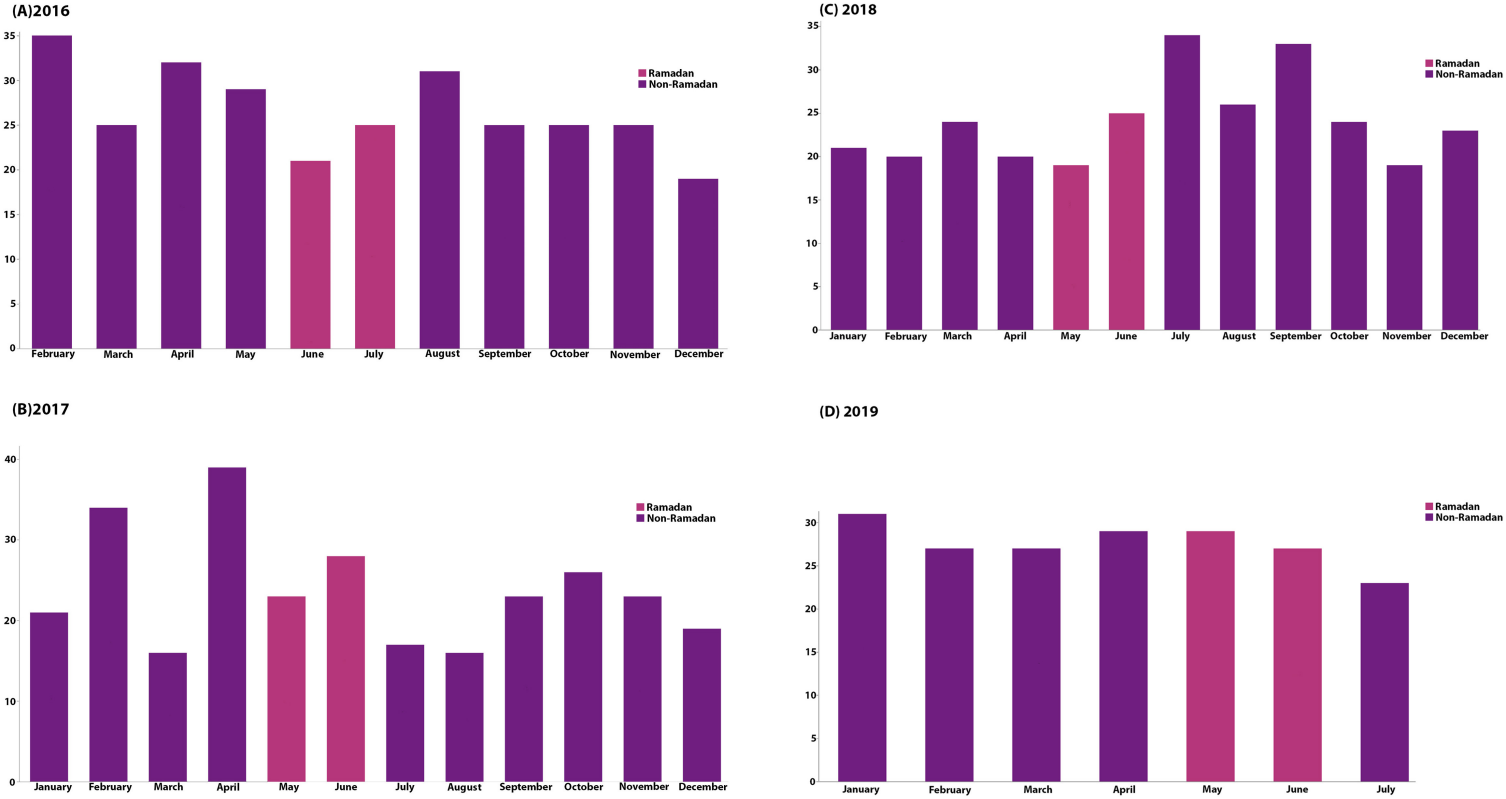
Demonstrates the number of IS cases by the Gregorian months; the different colors in each year represent Ramadan cases.

There was a statistically significant difference in ICU admissions (*p* = 0.009). ICU admissions were higher during Ramadan than in the non-Ramadan group (17.3 and 9%, respectively). However, there was no change in in-hospital stay and in-hospital mortality (*p* = 0.9 and *p* = 0.8, respectively). Utilization of tPA or endovascular thrombectomy was similar between the two groups (*P* = 0.8 and 0.2, respectively). Upon discharge, the NIHSS score was higher [mean 8 ± 9, during Ramadan vs. mean 5 ± 6, non-Ramadan group (*p* = *0.0045*)], but there was no difference in mean dependency at discharge and mRS score (*p* = 0.4 and 0.5, respectively). At 3 months, no difference was found regarding recurrent stroke or TIA (*p* = 0.8), see Table 2. In a gender-specific analysis during the Ramdan group in Table 3, smoking as a risk factor showed a significant difference, more prominent in males (*p* = 0.0061). Also, UTIs were more common in females (*p* = 0.005). However, no differences were found regarding the mean age, laboratory results, management options, complications, and outcomes. The distribution of Ramadan cases in comparison to other Hijri months is demonstrated in Figure 1. The highest number of Ramadan cases was noted in 1,438. Figure 2 demonstrates the distribution of Ramadan cases by the Gregorian months.

**Table 2:** Clinical characteristics of ischemic stroke (IS) patients in Ramadan months vs. non-Ramadan months.

**Characteristics**	**Non-Ramadan (*****N*** = **960)**	**During Ramadan (*****N*** = **98)**	***p***
Mean age (years) (± SD)	61 ± 12	59 ± 13	0.08
**Gender, *n* (%)**			
Male	658 (68.5)	56 (57.1)	*0.02*
Female	302 (31.5)	42 (42.9)	
**Medical history, *n* (%)**			
Ischemic heart disease	103 (10.7)	16 (16.3)	0.1
Arterial hypertension	702 (73.1)	66 (67.3)	0.2
Diabetes mellitus	634 (66.4)	63 (64.3)	0.7
Dyslipidemia	300 (31.2)	35 (35.7)	0.3
Atrial Fibrillation	70 (7.2)	10 (10.2)	0.3
History of smoking	163 (16.9)	13 (13.3)	0.3
History of ischemic stroke or TIA	250 (26)	19 (19.3)	0.1
**Mean NIHSS score upon admission (SD)**	7 (6)	8 (6)	0.09
**Mean modified Rankin scale (mRS) at admission (SD)**	0 ± 1	0 ± 1	*0.03*
**Laboratory results upon admission**			
Mean random blood glucose (mmol/L) (SD)	10.7 (5)	10.4 (5)	0.5
Mean sodium (mmol/L) (SD)	136 ± 4	136 ± 3	0.9
Mean creatinine (μmol/L) (SD)	94 ± 6	81 ± 27	*0.0005*
Mean blood urea nitrogen (mg/dL) (SD)	6.5 ± 4	5 ± 2	*0.0002*
**Management, *n* (%)**
Thrombolysis (t-PA)	82 (8.5)	8 (8.2)	0.8
Endovascular thrombectomy	38 (3.9)	1 (1)	0.2
**Median days of admission (IQR)**	6 (12)	6 (15)	0.9
**Complications, *n* (%)**
Pneumonia	48 (5)	6 (6)	0.6
Deep vein thrombosis/pulmonary embolism	13 (1.3)	1 (1)	0.7
Urinary tract infection	67 (6.9)	6 (6.1)	0.7
**Outcomes**
Intensive care unit (ICU) admission, *n* (%)	87 (9)	17 (17.3)	*0.009*
Mean NIHSS score upon discharge (SD)	5 (6)	8 (9)	0.0045
Mean modified Rankin scale (mRS) upon discharge (SD)	2 (2)	2 (2)	0.5
Mean dependency upon discharge (SD)	72 (32)	75 (28)	0.4
In-hospital mortality, *n* (%)	64 (6.6)	7 (7.1)	0.8
Recurrent stroke or TIA at 3 months, *n* (%)	5 (8.3)	0	0.8

*The differences between frequencies were investigated using a chi-square test. Continuous variables were tested using the T-test, ANOVA, and Kruskal-Wallis test. The bold values showed statistical significance (A p < 0.05 was considered significant). The P-value shows the statistical significance as obtained from the above tests*.

**Table 3:** Clinical characteristics and outcomes of ischemic stroke (IS) patients during Ramadan months based on gender.

**Characteristics**	**Male (*****N*** = **56)**	**Female (*****N*** = **42)**	***p***
Mean age (years) (SD)	60 (12)	57 (14)	0.8
**Medical history, *n* (%)**
Ischemic heart disease	9 (16)	7 (16.6)	0.9
Arterial hypertension	38 (67.8)	28 (66.6)	0.9
Diabetes mellitus	35 (62.5)	28 (66.6)	0.6
Dyslipidemia	19 (33.9)	16 (38)	0.6
Atrial Fibrillation	3 (5.3)	7 (16.6)	0.06
History of smoking	12 (21.4)	1 (2.3)	*0.0061*
History of ischemic stroke or TIA	11 (19.6)	8 (19)	0.9
**Mean NIHSS score upon admission (SD)**	8 (7)	8 (6)	0.09
**Mean modified Rankin scale (mRS) at admission (SD)**	0 (1)	0 (1)	0.3
**Laboratory upon admission**
Mean random blood glucose (mmol/L) (SD)	10.7 (5.3)	10.4 (5.2)	0.5
Mean sodium (mmol/L) (SD)	136 (4)	136 (3)	0.4
Mean creatinine (μmol/L) (SD)	84 (32)	77 (19)	0.9
Mean blood urea nitrogen (mg/dL) (SD)	5 (2)	5 (2)	0.8
**Management, *n* (%)**
Thrombolysis (t-PA)	4 (7.1)	4 (9.5)	0.6
Endovascular thrombectomy	1 (1.8)	0	0.2
**Median days of admission (IQR)**	6 (15)	6 (15)	0.6
**Complications, *n* (%)**
Pneumonia	2 (3.5)	4 (9.5)	0.2
Deep vein thrombosis/pulmonary embolism	0	1 (2.3)	0.4
Urinary tract infection	0	6 (14.29)	*0.005*
**Outcomes**
Intensive care unit (ICU) admission, n (%)	8 (14.2)	9 (21.4)	0.3
Mean NIHSS score upon discharge (SD)	9 (8)	8 (10)	0.4
Mean modified Rankin scale (mRS) upon discharge (SD)	2 (2)	2 (2)	0.6
Mean dependency upon discharge (SD)	76 (27)	74 (30)	0.5
In-hospital mortality, *n* (%)	3 (5.3)	4 (9.5)	0.4
Recurrent stroke or TIA at 3 months, *n* (%)	5 (8.3)	0	0.8

*The differences between frequencies were investigated using a chi-square test. Continuous variables were tested using the T-test, ANOVA, and Kruskal-Wallis test. The bold values showed statistical significance (A p < 0.05 was considered significant). The P-value shows the statistical significance as obtained from the above tests*.

## Discussion

This paper aimed to study ischemic stroke patients' clinical characteristics and outcomes during Ramadan vs. non-Ramadan months. We found an apparent gender difference between the two groups (non-Ramadan and Ramadan). There were no differences in vascular risk factors or laboratory results, except for BUN and creatinine. Regarding the outcomes, more ICU admissions and higher NIHSS upon discharge were observed during Ramadan. In gender-based analysis, smoking was a more prominent risk factor among males than UTIs, which were more common in females.

Although Ramadan carries a change in lifestyle, including dietary habits and physical activity, this did not affect our population's overall incidence of stroke. This conclusion seemed similar to most other studies exploring intermittent fasting (IF) and stroke (7–9, 11–13). However, fewer studies found a difference when comparing Ramadan with non-Ramadan months (6, 14). The latter studies suggested that Ramadan fasting was associated with an increased incidence of stroke. Also, cerebral venous and sinus thrombosis (CVT) seemed to increase during Ramadan in one study reported in Iran, in contrast to arterial thrombosis, in which most of the studies found no difference (15). Finally, it is essential to mention that several studies on blood pressure, HbA1c, and lipids profile reported improved parameters during Ramadan (2–4). However, this does not significantly affect stroke or risk factors during Ramadan months.

Ramadan seemed to have different gender distribution of IS patients compared to the rest of the year, which could be attributed to population-specific factors since other studies did not show this variation (7–9, 12, 13). Smoking appeared to be a significant risk factor during Ramadan among males in our study. This could explain why males are more prone to IS during Ramadan. Also, estrogen plays an important role in vascular risk factors (16). Studies showed that postmenopausal women carry a higher risk of HTN than premenopausal or perimenopausal women, which was attributed to the effect of estrogen (16). In addition, postmenopausal women have higher systolic BP than age-matched males with similar diastolic BP (16). In our study, the females' mean age during Ramadan indicates that most of our sample sizes were post-menopausal women, who could have increased their risk of higher blood pressure and, therefore, IS. Nevertheless, these gender differences in our population need to be explored further in future studies, as they could be significant. In the general analysis, no difference was observed in risk factors between Ramadan and non-Ramadan months. Similarly, other studies had no variations concerning stroke risk factors between the two groups (8, 9, 13). Furthermore, no difference was found between Ramadan and non-Ramadan months regarding cardiovascular events or risk factors (3, 11, 17). Also, one study suggests that Ramadan fasting is associated with an improvement in 10-year coronary heart disease risk scores (2). Although Ramadan fasting leads to dehydration during long periods, surprisingly, BUN and creatinine were higher during non-Ramadan periods. The difference in the sample size could explain this. However, other metabolic labs (sodium, creatinine, random glucose) did not show any significant difference. One other study included BUN and showed that it is higher during Ramadan than before, inconsistent with our findings (12). The rest of the laboratory findings in the literature were similar to our findings. (6, 8, 12, 13).

Western diet can delay and impair the recovery of neurons following injury through exacerbation of neuroinflammation, potentially through several pathways, including gut dysbiosis, epigenetic modulation, and induction inflammatory pathways (18). On the other hand, several studies suggest that brain cell adaptation to ischemia and recovery of neurons following ischemic insult is more pronounced with IF (19–24). The proposed mechanism by which IF can improve neuron adaptation includes blunting cell death and limiting the pathological increase in cell proliferation (19). Furthermore, IF reduces oxidative stress and inflammatory pathways following IS, which is usually due to upregulation in multiple neuroprotective factors, including neurotrophic factors, protein chaperones, antioxidant enzymes, and downregulation of pro-inflammatory cytokines (20, 22). It was also found that the IF diet aids the expression of genes that are essential for plasticity and regeneration (22). This, however, was not evident in our findings since ICU admission and NIHSS scores were higher during Ramadan. Upon admission and during the hospital stay, patients in our study showed no differences in pneumonia, UTI, or DVT/PE, which are the complications we explored. This was expected since Ramadan fasting is not associated with increased frequency and length of hospital stay (25).

Nevertheless, we found that ICU admissions tend to increase during Ramadan. Moreover, the NIHSS score upon admission was similar between Ramadan and non-Ramadan months. An Egyptian study found that the NIHSS score upon admission is higher during Ramadan when compared to the months before and after Ramadan. However, the Israel study found no difference in NIHSS score, consistent with our findings (6). Ramadan fasting was not associated with alteration of management options used in stroke, as the frequency of utilizing tPA or thrombectomy remained the same. There was no significant difference regarding in-hospital mortality, though, and high NIHSS scores upon discharge were evident during Ramadan.

In contrast to our findings, the Egyptian study demonstrated that mortality was significantly higher during Ramadan than before or after Ramadan. However, the Israeli study did not find any difference in mortality. These differences in outcomes could be attributed to environmental factors, as they could influence stroke risk factors (26). Despite high NIHSS scores found upon discharge, no difference was observed in the patients' dependency. Furthermore, our study did not show any differences in ischemic stroke recurrence or TIA at 3 months. This is especially important as the effect of these metabolic changes during Ramadan might last since the result is not immediate.

Our main findings were consistent with those of most other studies on the same topic, which reported no significant difference in ischemic stroke incidence during Ramadan (7–9, 11–13). However, our study was limited since we did not include hemorrhagic strokes due to insufficient numbers. Also, this was a single-center and retrospective study. Moreover, since it is difficult to determine the fasting situation in the setting of acute stroke, we are not certain about the fasting situation of the patients. However, the study is conducted in an Islamic country during Ramadan, during which almost all Muslims are fasting. Lastly, it was difficult to compare the Ramadan vs. non-Ramadan cases statistically due to the small sample size number. Therefore, we recommend further research, preferably multicenter, from different regions with a larger sample size, including hemorrhagic and ischemic strokes, to understand this topic better.

## Conclusion

In conclusion, Ramadan fasting has no effect on the rate of ischemic stroke during Ramadan. Also, findings did not vary regarding the duration of hospital stay and complications. However, ICU admissions and NIHSS scores upon discharge were higher during Ramadan.

## Data availability statement

The raw data supporting the conclusions of this article will be made available by the authors without undue reservation.

## Author contributions

NA and MAld wrote the introduction and results and helped with statistical analysis. BA and MAld did the statistical analysis. MAld, MAlh, SA, WA, FA, AAld, and MAls collected the data, reviewed the literature, and co-wrote the results. NA and AAl reviewed and edited the final manuscript. NA wrote the discussion and reviewed the literature. All authors contributed to the article and approved the submitted version.

## Conflict of interest

The authors declare that the research was conducted without any commercial or financial relationships construed as a potential conflict of interest.

## Publisher's note

All claims expressed in this article are solely those of the authors and do not necessarily represent those of their affiliated organizations, or those of the publisher, the editors and the reviewers. Any product that may be evaluated in this article, or claim that may be made by its manufacturer, is not guaranteed or endorsed by the publisher.
